# Brain atrophy and patch-based grading in individuals from the CIMA-Q study: a progressive continuum from subjective cognitive decline to AD

**DOI:** 10.1038/s41598-019-49914-3

**Published:** 2019-09-19

**Authors:** Christine Marcotte, Olivier Potvin, D. Louis Collins, Sylvie Rheault, Simon Duchesne

**Affiliations:** 1 Centre de recherche CERVO Research Centre, Québec, Canada; 2 Montreal Neurological Institute, McGill University, Montreal, Canada; 3 True Positive Medical Devices Inc., Montreal, Canada; 4 Département de neurosciences, Université de Montréal, Montréal, Canada; 5 Centre de recherche de l’Institut universitaire de gériatrie de Montréal, Montréal, Canada; 6 Département de radiologie et médecine nucléaire, Faculté de médecine, Université Laval, Québec, Canada

**Keywords:** Predictive markers, Alzheimer's disease

## Abstract

It has been proposed that individuals developing Alzheimer’s disease (AD) first experience a phase expressing subjective complaints of cognitive decline (SCD) without objective cognitive impairment. Using magnetic resonance imaging (MRI), our objective was to verify whether SNIPE probability grading, a new MRI analysis technique, would distinguish between clinical dementia stage of AD: Cognitively healthy controls without complaint (CH), SCD, mild cognitive impairment, and AD. SNIPE score in the hippocampus and entorhinal cortex was applied to anatomical T1-weighted MRI of 143 participants from the Consortium pour l’identification précoce de la maladie Alzheimer - Québec (CIMA-Q) study and compared to standard atrophy measures (volumes and cortical thicknesses). Compared to standard atrophy measures, SNIPE score appeared more sensitive to differentiate clinical AD since differences between groups reached a higher level of significance and larger effect sizes. However, no significant difference was observed between SCD and CH groups. Combining both types of measures did not improve between-group differences. Further studies using a combination of biomarkers beyond anatomical MRI might be needed to identify individuals with SCD who are on the beginning of the clinical continuum of AD.

## Introduction

The pathophysiological cascade of Alzheimer’s disease (AD) is thought to begin decades before the diagnosis of clinical dementia^1^. Thus, being able to identify people in the preclinical stage of AD is fundamental if we are to develop and offer interventions that could potentially delay or decelerate its onset^2^.

Mild cognitive impairment (MCI) is considered such a prodromal stage of AD, whereby an objective cognitive decline is present without dementia^2,3^. A systematic review of 32 cohort studies following individuals with MCI reported a median annualized progression rate to the dementia stage of AD of 8.4%^4^. The studies with the longest follow-ups (9 and 10 years) observed that after this period of time, between 41 and 56% of the participants had progressed to dementia stage of AD. However, at the MCI stage, the brain seems to have already suffered such damage that an effective treatment to reverse neurodegeneration is hardly conceivable^5^.

It has since been proposed that pre-MCI individuals can be identified as they go through a phase expressing a subjective complaint of memory decline. This subjective cognitive decline (SCD) status, where cognitive performance is intact, has been associated with later progression to dementia^6–8^ and AD neuropathological manifestations^9^. In their biomarkers’ review on the topic, Sun *et al*.^10^ concluded that there is promising evidence that clinicians should be able to adequately differentiate pre-AD SCD and non-AD SCD based on the presence of pathophysiological biomarkers in cerebrospinal fluid (CSF) and neuroimaging, with the caveat that neuroimaging is still at an immature stage in terms of sufficiently sensitive and specific techniques.

Indeed, while comparative atrophy of brain structures (e.g. volumes, surfaces and thicknesses) as measured from magnetic resonance imaging (MRI) appears to be present in SCD^11^, differences between SCD and cognitively healthy controls without complaint (CH) are subtle^12^. Other, newer MRI analysis techniques drawing upon machine learning principles, such as probability grading via a nonlocal image patch estimator (SNIPE)^13^, might be more sensitive to these differences. In a 2015 study, Coupe *et al*.^14^ demonstrated that hippocampal SNIPE probability grading in CH yielded a prediction accuracy up to 72.5%, seven years before conversion to probable AD; significantly higher than hippocampal volumes (58.1%).

Our objective was to compare individuals on the clinical progression continuum of AD (CH, SCD, MCI, and AD) in terms of brain morphometry using both standard atrophy measures (cortical thicknesses, volumes, and surfaces) as well as SNIPE probability grading. For the latter, we specifically focused on the hippocampal (HPC) and entorhinal cortices (EC) since AD neuropathology is initially expected in those regions^15^. We hypothesized that for both EC and HPC there would be (1) gradients of increasing biomarker severity from CH to AD, and (2) differences between SCD and CH, with SNIPE scores showing larger differences than atrophy measures. We further hypothesized that other limbic structures (e.g. medial and lateral temporal lobe) would gradually become affected as individuals experience further objective cognitive decline.

## Methods

### Participants

The data used in this article were obtained from the Consortium pour l’identification précoce de la maladie Alzheimer - Québec (CIMA-Q)^16^, founded in 2013 with a $2,500,000 grant from the *Fonds d’Innovation Pfizer - Fond de Recherche Québec – Santé sur la maladie d’Alzheimer et les maladies apparentées*. The main objective is to build a cohort of participants characterized in terms of cognition, neuroimaging and clinical outcomes in order to acquire biological samples allowing (1) to establish early diagnoses of Alzheimer’s disease, (2) to provide a well characterized cohort and (3) to identify new therapeutic targets. The principal investigator and director of CIMA-Q is Dr Sylvie Belleville from the Centre de recherche de l’Institut universitaire de gériatrie de Montréal, CIUSSS Centre-sud-de-l’Île-de-Montréal. CIMA-Q represent a common effort of several researchers from Québec affiliated to Université Laval, Université McGill, Université de Montréal, et Université de Sherbrooke. CIMA-Q recruited 350 cognitively healthy participants, with subjective cognitive impairment, mild cognitive impairment, or Alzheimer’s disease, between 2013–2016. Volunteers were recruited from memory clinics, through advertisements posted in the community and amongst participants from the NuAge population study^17^.

Inclusion criteria were:

1. Being 65 years old and over
2. Living in the community or residence for an independent person
3. Have a score on the telephone-mini mental state examination^18^ (T-MMSE) of 17 or higher
4. Understand, read and write French or English
5. Have sufficient visual and auditory acuity to be able to go through the neuropsychology tests visit.
6. For participants with mild AD, be accompanied during clinical visits. For other participants, have an informant to answer questions (in person or by phone or in writing).
7. Be willing to: answer questionnaires about one’s state of health; have a physical and neuropsychological evaluation; submit to a blood test.

Exclusion criteria were

1. Plan to move out of Quebec in the next three years
2. Have a score of 20 or higher (≥20/27) on the Patient Health Questionnaire-9 (PHQ-9) scale.
3. Have a score on the Clinical dementia rating(CDR) greater than 1 (>1).
4. Have a disease or impairment of the central nervous system, including:

- Subdural hematoma (active or past)
- Subarachnoid hemorrhage (active or past) • Primary or metastatic brain cancer
- Epilepsy (active)
- Dementia (other than mild Alzheimer’s disease) or other neurodegenerative diseases

5. Have had intracranial surgery
6. Have an active addiction to alcohol, drugs or narcotics.
7. Have regular consumption of benzodiazepines greater than the equivalent of 1 mg per day lorazepam taken orally.
8. Have an illness/condition that is related to cognitive impairment or one that could interfere with the subject’s participation in the project.

We based our analyses on the 143 participants from CIMA-Q study who underwent MRI. Ethics approval was obtained from the Institutional Review Board of the Institut universitaire de gériatrie de Montréal. All research was performed in accordance with relevant guidelines/regulations and informed consent was obtained from all participants at entry in the study. Participants were classified into four groups according to criteria by the National Institute on Aging and Alzheimer’s Association (NIA-AA)^19,20^ and the Subjective Cognitive Decline Initiative Working Group^21,22^: cognitively healthy (CH, n = 30), simple subjective cognitive decline (SCD, n = 67), amnestic mild cognitive impairment (MCI, n = 30), and probable Alzheimer’s disease (AD, n = 16). Furthermore, early (eMCI) and late (lMCI) MCI were distinguished based on their MoCA and episodic memory performance (logical memory test). MCI could be single or multiple domains. Specific criteria for each category are presented in Table 1.

**Table 1:** Criteria used to classify participants into AD, lMCI, eMCI, SCD and CH groups.

Group	Subjective memory decline^a^	MoCA	CDR	Logical memory test	
				*Education*	*Score*
AD^b^	Yes, but this does not worry me OR Yes, this worries me	13–25 (inclusively)	1.0	≥16 years 8–15 years 0–7 years	≤8 ≤ 4 ≤ 2
lMCI^c^	Yes, but this does not worry me OR Yes, this worries me	20–25 (inclusively)	0.5	≥16 years 8–15 years 0–7 years	≤8 ≤ 4 ≤ 2
eMCI^c^	Yes, but this does not worry me OR Yes, this worries me	20–26 (inclusively)	0.5	≥16 years 8–15 years 0–7 years	9–11 5–9 3–6
SCD	Yes, this worries me	≥26	0	≥16 years 8–15 years 0–7 years	≥9 ≥ 5 ≥ 3
CH	No OR				
Yes, but this does not worry me	≥26	0	≥16 years 8–15 years 0–7 years	≥9 ≥ 5 ≥ 3	

^a^Based on the following question: “Do you feel like your memory is becoming worse?”^52^.

^b^Fit the probable AD clinical core criteria of the National Institute on Aging and Alzheimer’s Association^19^.

^c^Fit the MCI clinical core criteria of the National Institute on Aging and Alzheimer’s Association^20^.

*Note*. MoCA: Montreal Cognitive Assessment; CDR: Clinical Dementia Rating Scale.

### Image acquisition and processing

T1-weighted MRI scanning was performed at five different sites (see Table 1). Scans were acquired from either Siemens Healthcare (TrioTim and Prisma Fit) or Philips Medical Systems (Achieva and Ingenia) scanners with a magnetic field strength of 3 Tesla. All scans were first visually inspected by a rater blinded to the diagnosis. Six participants were excluded due to poor quality scans (CH = 1, SCD = 1, AD = 4). The final sample included 137 participants (CH = 29, SCD = 66, eMCI = 22, lMCI = 8, AD = 12).

### Atrophy state measurements

To derive morphometry measurements for the purpose of computing atrophy states, we performed preprocessing and segmentation with the *FreeSurfer* (http://freesurfer.net) image analysis suite (version 5.3), using recon-all with default parameters. The technical details of these procedures are described in prior publications^23,24^. We used cortical morphometric measures generated from the Desikan-Killiany-Tourville atlas (aparc.DKTatlas40 files) and volumes from subcortical regions (aseg.stats file). The atrophy measure was obtained by adjusting morphometric measures for age, sex, estimated intracranial volume, scanner manufacturer and magnetic field strength according to normative values, expressing the final results as adjusted Z scores^25–27^, with negative values indicating smaller than expected values for healthy controls.

Segmentation accuracy was visually examined using 19 coronal slices and 5 axial slices. Scans with apparent segmentation errors were then inspected slice by slice using *FreeView* (http://freesurfer.net). Cortical regions with inadequate inclusion (e.g. dura mater) or omission of approximately 100 voxels or more were excluded from statistical analyses. The HPC and EC had no segmentation error. On a total of 78 regions, the mean (±SD) number of excluded regions per participant was 1.8 (CH: 1.6 ± 2.3; SCD: 1.3 ± 2.3; eMCI: 1.6 ± 1.8; lMCI: 4.6 ± 2.9; AD: 3.5 ± 3.2).

### Probability grading

SNIPE probability grading scores were provided under collaboration with True Positive Medical Device Inc. (TPMD, *AlzMETRIX*™). In this patented technique, images first undergo preprocessing, which included denoising based on an optimized nonlocal means filter^28^; correction of inhomogeneities using N3^29^; registration to stereotaxic space using ICBM152 template linear transform (1 × 1 × 1 mm^3^ voxel size)^30^ using a template derived from the Alzheimer’s Disease Neuroimaging Initiative (ADNI) study database^31^; linear intensity normalization of each subject on template intensity; and brain extraction using BEaST^32^, based on patch-based segmentation^33,34^. Then HPC and EC SNIPE grading scores^35,36^ are obtained by estimating the nonlocal similarity of the subject’s image to training samples of healthy aging subjects and participants with AD MRI. For each voxel in a new subject to be analyzed, the method defines a 7 × 7 × 7 voxel patch centered on the voxel. The procedure then searches the template library for similar patches. Template region labels are weighted by patch similarity, and the region label with the maximum weight is then associated with the voxel. At the same time, the template group (1 for healthy controls and 2 for AD subjects) is also weighted by the patch similarity. The resulting average weight is used as a grading value to indicate how similar this voxel is to the training AD group (for details see^36^). The SNIPE grading score is the average of the voxels within a region (e.g., HPC or EC). Normative SNIPE Z scores adjusted for age and sex were computed from healthy participants in a normative database internal to TPMD.

### Cognitive and behavioral assessments

Cognitive functioning was assessed using the Montreal Cognitive Assessment (MoCA)^14^ and the Logical Memory I subtest of the Wechsler Memory Scale third edition^37^. Anxiety and depressive symptoms were assessed using the Geriatric anxiety inventory (GAI)^38^ and Geriatric depressive scale (GDS)^39^.

### Statistical analyses

Participants’ characteristics were compared between groups using one-way ANOVA and Chi-square. For all regions, differences between CH and SCD in terms of surface, thickness and volumes were estimated by ANOVA with false discovery rate (FDR) *p* value correction to adjust for multiple comparisons^40^. We used Cohen’s *d* to quantify effect size. To verify whether a combination of regions and measures, rather than a single metric, could highlight larger differences between groups, composite scores for atrophy and SNIPE score were computed and tested. Composite scores were either a) the mean of all regions; b) the mean of both bilateral SNIPE measures; or c) the combination of bilateral atrophy and SNIPE score means.

Differences for the main outcomes (HPC and EC measures) were assessed using one-way ANOVAs with post-hoc Tukey’s tests for pairwise comparisons. To assess whether anxiety and depressive symptoms influenced the association between biomarkers and cognitive complaints, using linear regression, we tested the interaction terms between GAI/GDS scores and cognitive complaints (CH vs SCD) on the prediction of biomarkers.

All statistical analyses were conducted in Python using SciPy^41^ and StatsModels^42^ modules.

## Results

### Participants characteristics

Table 2 shows the characteristics of the participants. Age (*p* = 0.088) and sex (*p* = 0.963) did not significantly differ between groups while, as expected, there was a significant difference between groups for MoCA scores (*p* < 0.001).

**Table 2:** Participants characteristics.

Characteristic	CH	SCD	eMCI	lMCI	AD
Age, mean(sd)	70 (6.3)	71 (6.4)	73 (8.4)	76 (6.9)	75 (7.7)
**Sex**, **(n)**					
Female	17	37	11	4	6
Male	12	29	11	4	6
GAI, mean(sd)	1.3 (2.5)	2.9 (3.5)	3.1 (4.6)	4.6 (7.5)	1.6 (2.5)
GDS, mean(sd)	2.2 (2.9)	5.6 (4.4)	6.6 (5.3)	7.1 (6.5)	4.6 (2.6)
MoCA, mean(sd)	28.0 (1.94)	27.7 (1.38)	25.2 (1.82)	23.1 (3.09)	17.2 (5.36)
RAVLT, mean(sd)	11.2 (2.3)	9.5 (2.7)	7.9 (3.1)	3.4 (2.4)	0.6 (1.2)

AD: Alzheimer’s disease, CH: healthy controls, eMCI: early GAI: Geriatric Anxiety Inventory, MCI, GDS: Geriatric Depression Scale, lMCI: late MCI, MCI: mild cognitive impairment, MoCA: Montreal Cognitive Assessment, RAVLT: Rey Auditory Verbal Learning Test delayed recall score, SCD: subjective cognitive decline.

### HPC and EC atrophy state

**Figure 1:**
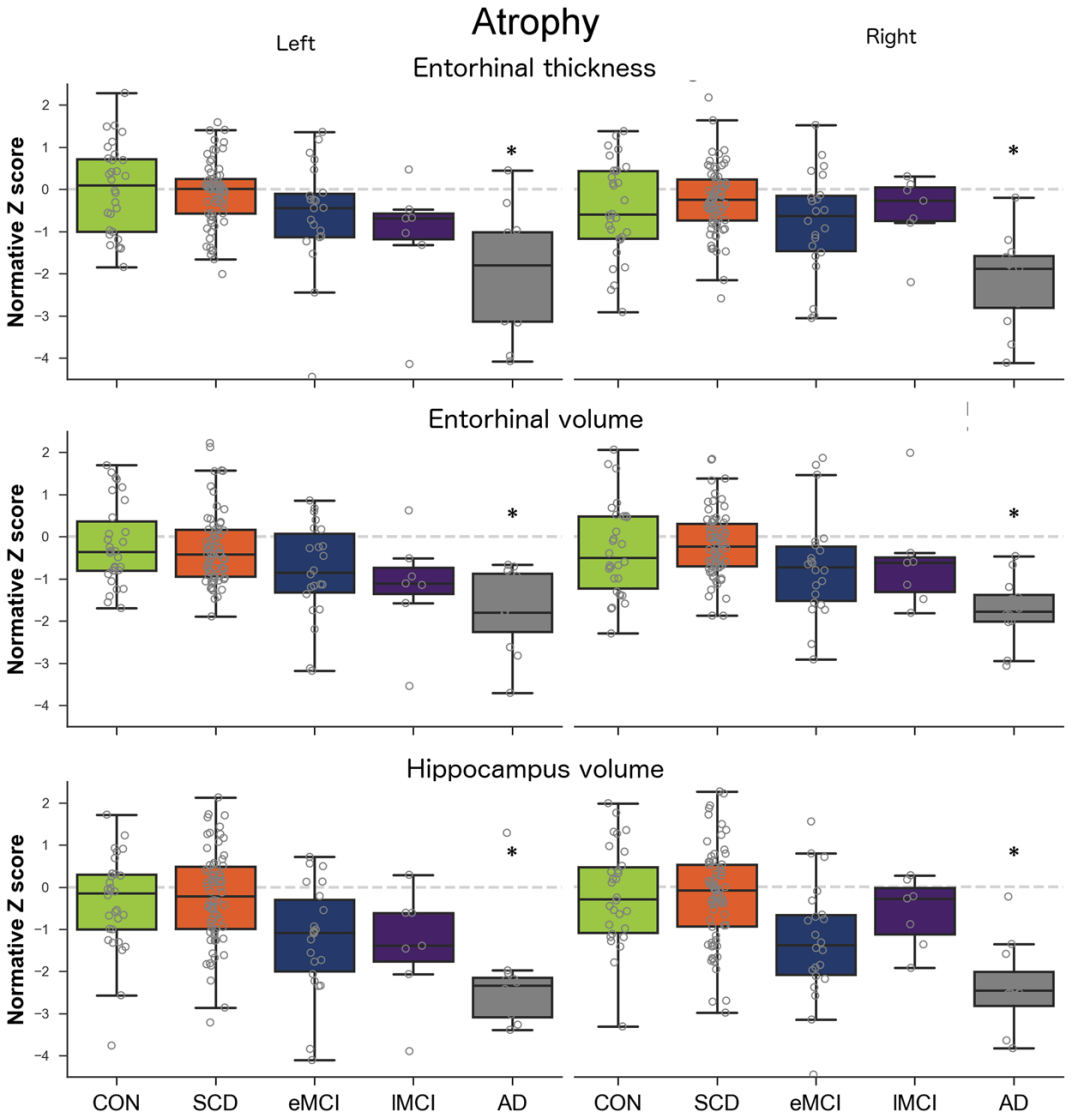
Atrophy measures for the left and right entorhinal cortex and across groups. AD: Alzheimer’s disease, CH: healthy controls, eMCI: early MCI, lMCI: late MCI, MCI: mild cognitive impairment, SCD: subjective cognitive decline. Dashed grey line represents normality (Z score of 0). Stars indicate significant difference compared to CH group.

Figure 1 shows atrophy states for the HPC and EC for each group. Qualitatively, atrophy results displayed a gradient of changes from CH to AD in the EC and HPC, especially for the left EC and HPC. However, only the AD group (left and right *p* < 0.001) had significantly lower EC and HPC volumes and lower EC thickness than CH. Furthermore, only the left EC volume and thickness were higher in CH than in SCD, with very weak effect sizes (*d*: 0.10 for both).

### HPC and EC probability grading

**Figure 2:**
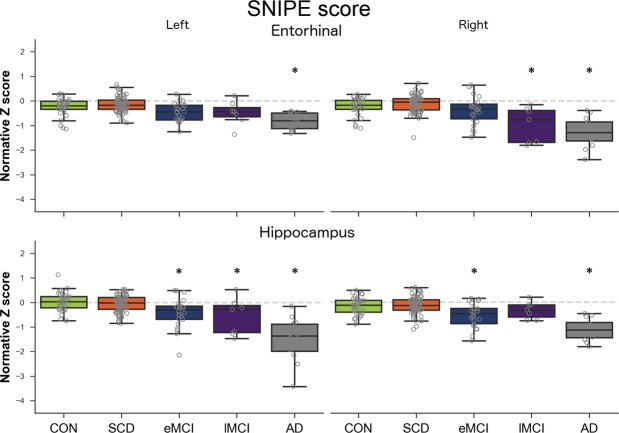
SNIPE scores for left and right entorhinal cortex and hippocampus across groups. AD: Alzheimer’s disease, CH: healthy controls, eMCI: early MCI, lMCI: late MCI, MCI: mild cognitive impairment, SCD: subjective cognitive decline. Dashed grey line represents normality (Z score of 0). Stars indicate significant difference compared to CH group.

SNIPE score results (Fig. 2) were similar to atrophy results, with a gradient of changes from CH to AD. For the HPC, all groups significantly differed from the CH group on either left or right (eMCI left *p* = 0.009, right *p* = 0.004; lMCI left *p* = 0.037, right *p* = 0.760), except the SCD group (left and right *p* = 0.900). For the EC, only AD (left *p* < 0.001, right *p* < 0.001) and lMCI (left *p* < 0.001, right *p* = 0.480) significantly differed from the CH group. Only the left HPC score was higher in CH than SCD, with a limited effect size (*d*: 0.25).

### Composite scores

**Figure 3:**
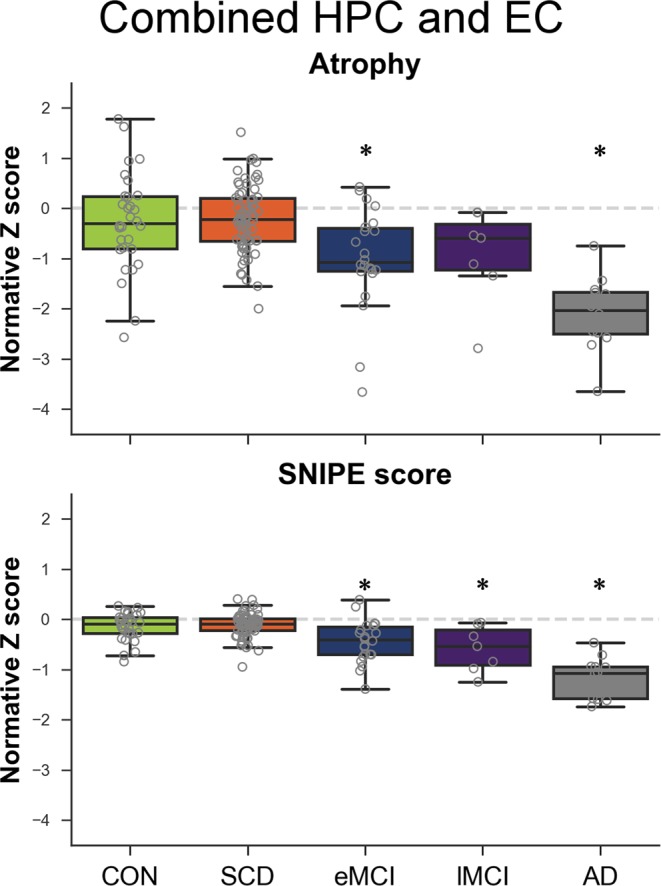
Composite atrophy and SNIPE scores of bilateral entorhinal cortex and hippocampi across groups. AD: Alzheimer’s disease, CH: healthy controls, eMCI: early MCI, lMCI: late MCI, MCI: mild cognitive impairment, SCD: subjective cognitive decline. Dashed grey line represents normality (Z score of 0). Stars indicate significant difference compared to CH group.

Figure 3 presents the results for the combined HPC-EC scores. AD (*p* < 0.001) and eMCI (*p* = 0.031) had significantly lowered composite atrophy score from the CH group, but not SCD (*p* = 0.900) and lMCI (*p* = 0.316). All groups had lower composite SNIPE score than the CH group (AD *p* < 0.001, eMCI *p* = 0.006, lMCI *p* = 0.008), except the SCD group (*p* = 0.900). Combining atrophy and SNIPE composite score did not improve group differences and did not yield a difference between CH and SCD groups (*p* = 0.900).

The largest effect size between CH and AD were for the composite SNIPE score (*d*: 2.97), and the right (*d*: 2.41) and left (*d*: 2.23) HPC. Effect sizes for atrophy measures were lowered (composite *d*: 2.09, right HPC volume *d*: 1.59, left EC volume *d*: 1.58).

### Other brain areas atrophy state

**Figure 4:**
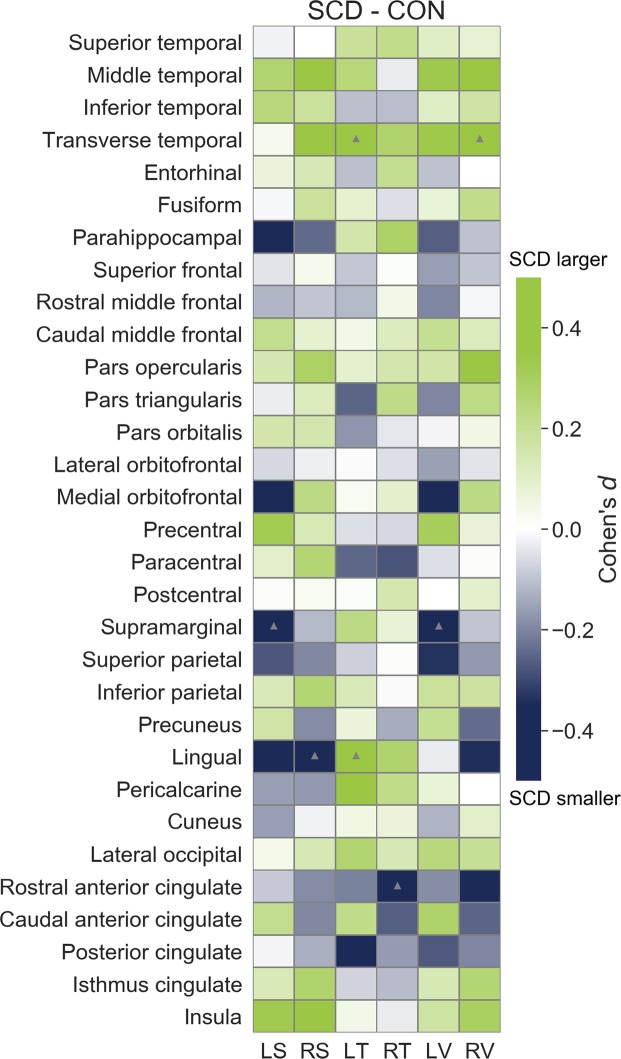
Effect sizes between SCD and CH groups for cortical surface, thickness and volume. LS = Left surface, RS = Right surface, LT = Left thickness, RT = Right thickness, LV = Left volume, RV = Right volume. Grey triangles indicate *p* < 0.05 value before multiple correction.

**Figure 5:**
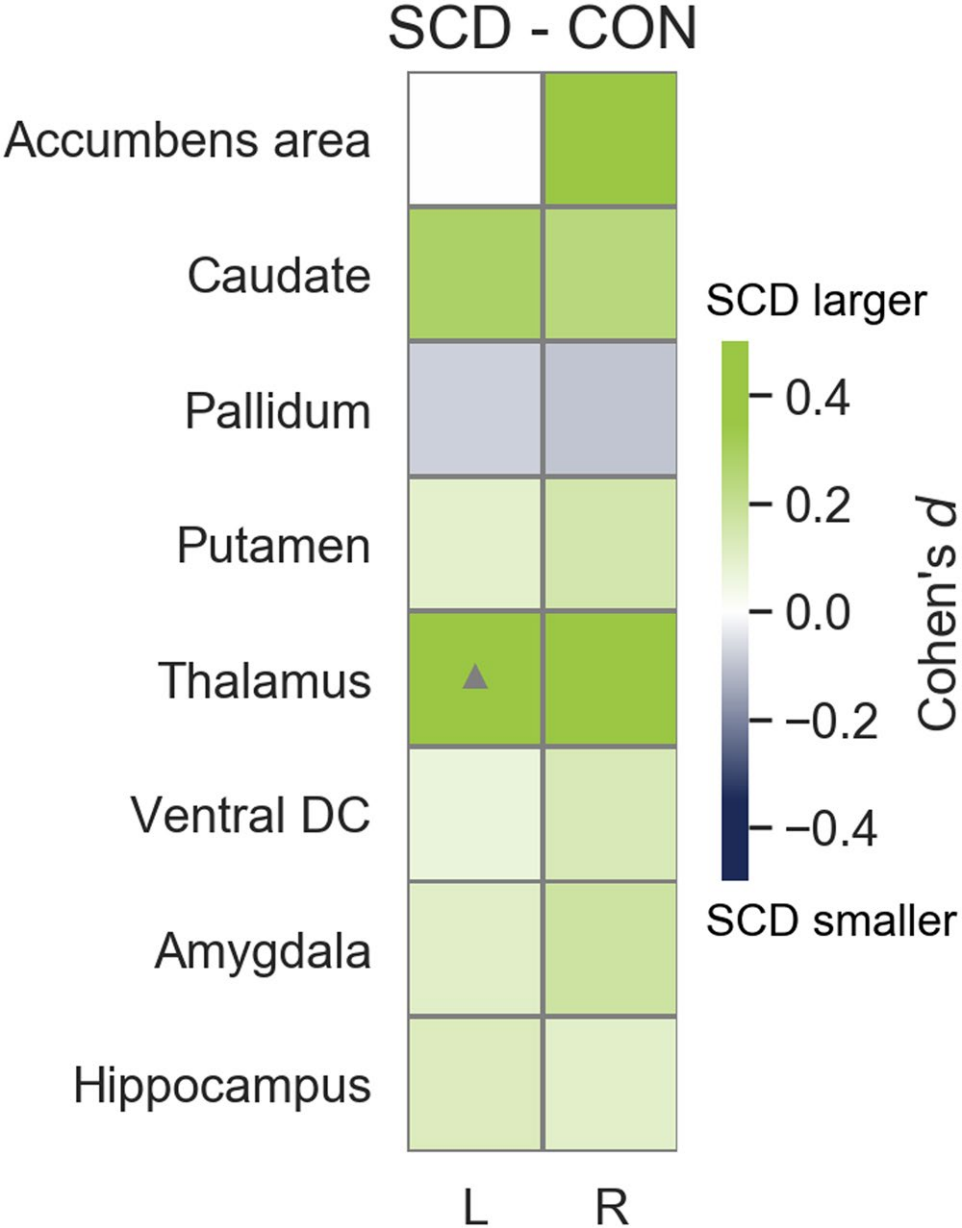
Effect sizes between SCD and CH groups for subcortical volumes. Note. L = Left, R = Right. Grey triangles indicate *p* < 0.05 value before multiple correction.

Figures 4 and 5 show the effect sizes between SCD and CH groups for all atrophy measures. We found eight significant differences, four measures were smaller (left supramarginal surface and volume, right rostral anterior cingulate thickness, and right lingual surface) and four measures were larger (transverse temporal left thickness and right surface, left lingual thickness, and left thalamus volume) in SCD than in CH. However, none of those significant differences remained after FDR correction for multiple comparisons.

### Impact of co-morbidities

Anxiety and depressive symptoms had no significant influence on the relationships between cognitive complaints and SNIPE or atrophy scores (*p* > 0.05).

## Discussion

The objective of this study was to compare individuals on the clinical progression continuum of AD on standard brain morphometry measures (volumes, surfaces and thicknesses) as well as a more recent, machine-learning derived technique for probability grading named SNIPE score.

Since hippocampal SNIPE score has been shown to predict dementia stage of AD in CH 7 years before conversion (72.5% accuracy), we expected SNIPE measures of the HPC and EC to highlight subtle differences between CH and SCD that could not be identified using usual morphometric measures. While the results confirmed our hypothesis of gradients of increasing biomarker severity from CH to clinical AD, they revealed no significant differences between CH and SCD. Notwithstanding, SNIPE measures appeared to be moderately more sensitive to dementia stage of AD since differences between groups reached a higher level of significance, and larger effect sizes between AD and CH were observed compared to standard atrophy measures of cortical thickness or structure volume.

Moreover, the combination of SNIPE score and standard atrophy measures did not highlight any group differences. Combining morphometry measures with other biomarkers should have improved highlighting very early differences on the clinical AD spectrum. However, the early preclinical nature of the disease makes the grey matter alteration very subtle and, consequently, very hard to detect.

In fact, as reported in previous studies^43,44^, cellular neurodegeneration and cognitive decline are more spatially and temporally related to neurofibrillary tangles (NFT), composed of tau proteins, than to amyloid plaques. While the choice of EC and HPC regions for SNIPE score calculation was justified as being early targets of tau accumulation, future studies should conduct probability grading in the transentorhinal cortex, given that the pattern of NFT propagation has been reported to initiate first in this region, then to the entorhinal cortex^15,45^. This may explain in part the lack of sensitivity of SNIPE score to differentiate between SCD and CH. However, at the moment no automated method seems available to segment the transentorhinal region, and segmentations thus have to be conducted manually. Alternatively, changes in the SCD group may be at an undetectable level, and dysregulation of long term potentiation or synaptic losses^46^ could justify memory complaints, before neuronal death and quantifiable atrophy. This subtends the need and importance to follow this cohort longitudinally to assess the positive and negative predictive value of SNIPE score, as well as putative thresholds for baseline assessment that may be indicative of future decline.

While SCD, essentially defined by subjective memory decline, appears to be a promising feature to identify future cognitive decline related to AD, it is a relatively generic symptom encompassing a heterogeneous group of individuals since it is not specific to AD pathology, nor to AD symptomatology. First, a recent and large study^9^ confirmed that SCD was associated with higher risks of incident of MCI (OR: 1.19) and AD (OR: 1.64), but also revealed that it increases the risk of AD pathological diagnostic (OR: 1.96). However, SCD is also associated with Lewy bodies (OR: 2.47), TDP-43 (OR: 1.33), hippocampal sclerosis (OR: 2.90), and amyloid angiopathy (OR: 1.46). Indeed, in addition to hippocampal sclerosis^47^, Lewy bodies^48^ and TDP-43^49^ are common phenomenon observed in the medial temporal lobe that have been shown to correlate with memory performance^47,48^. Secondly, SCD is unspecific to symptomatology, since it is related to multiple other symptoms, especially psychiatric symptoms. For example, it is well-documented that SCD is related to anxiety and depression^50,51^. However, in the present study, we showed that anxiety and depressive symptoms did not explain the lack of difference between CH and SCD.

Compared to standard atrophy measures, SNIPE score appeared slightly more sensitive to differentiate the dementia stage of AD, but not sufficiently to differentiate between SCD and CH.

Further studies using a combination of biomarkers beyond anatomical MRI might be needed to identify pathophysiological biomarkers of SCD.

## Data Availability

The CIMA-Q dataset is publicly available at http://www.cima-q.ca.

## References

[1] Morris JC (2005). Early-stage and preclinical Alzheimer disease. Alzheimer disease and associated disorders.

[2] Sperling RA (2011). Toward defining the preclinical stages of Alzheimer’s disease: recommendations from the National Institute on Aging-Alzheimer’s Association workgroups on diagnostic guidelines for Alzheimer’s disease. Alzheimer’s & dementia: the journal of the Alzheimer’s Association.

[3] Petersen RC (2004). Mild cognitive impairment as a diagnostic entity. J Intern Med.

[4] Ward A, Tardiff S, Dye C, Arrighi HM (2013). Rate of conversion from prodromal Alzheimer’s disease to Alzheimer’s dementia: a systematic review of the literature. Dementia and geriatric cognitive disorders extra.

[5] Cheng YW, Chen TF, Chiu MJ (2017). From mild cognitive impairment to subjective cognitive decline: conceptual and methodological evolution. Neuropsychiatr Dis Treat.

[6] Jessen F (2010). Prediction of dementia by subjective memory impairment: effects of severity and temporal association with cognitive impairment. Arch Gen Psychiatry.

[7] Reisberg B, Shulman MB, Torossian C, Leng L, Zhu W (2010). Outcome over seven years of healthy adults with and without subjective cognitive impairment. Alzheimers Dement.

[8] Mitchell AJ, Beaumont H, Ferguson D, Yadegarfar M, Stubbs B (2014). Risk of dementia and mild cognitive impairment in older people with subjective memory complaints: meta-analysis. Acta psychiatrica Scandinavica.

[9] Arvanitakis Zoe, Leurgans Sue E., Fleischman Debra A., Schneider Julie A., Rajan Kumar B., Pruzin Jeremy J., Shah Raj C., Evans Denis A., Barnes Lisa L., Bennett David A. (2018). Memory complaints, dementia, and neuropathology in older blacks and whites. Annals of Neurology.

[10] Sun Y, Yang FC, Lin CP, Han Y (2015). Biochemical and neuroimaging studies in subjective cognitive decline: progress and perspectives. CNS Neurosci Ther.

[11] Lista S (2015). Evolving Evidence for the Value of Neuroimaging Methods and Biological Markers in Subjects Categorized with Subjective Cognitive Decline. J Alzheimers Dis.

[12] Peter J (2014). Gray matter atrophy pattern in elderly with subjective memory impairment. Alzheimers Dement.

[13] Coupe P (2012). Scoring by nonlocal image patch estimator for early detection of Alzheimer’s disease. NeuroImage. Clinical.

[14] Coupe P (2015). Detection of Alzheimer’s disease signature in MR images seven years before conversion to dementia: Toward an early individual prognosis. Human brain mapping.

[15] Braak H, Alafuzoff I, Arzberger T, Kretzschmar H, Del Tredici K (2006). Staging of Alzheimer disease-associated neurofibrillary pathology using paraffin sections and immunocytochemistry. Acta Neuropathol.

[16] Belleville, S. *et al*. The Consortium for the Early Identification of Alzheimer’s Disease-Quebec (CIMA-Q). *Alzheimer’s & Dementia: Diagnosis*, *Assessment & Disease Monitoring* (in press). 10.1016/j.dadm.2019.07.003 PMC6880140 31788534

[17] Gaudreau P (2007). Nutrition as a determinant of successful aging: description of the Quebec longitudinal study Nuage and results from cross-sectional pilot studies. Rejuvenation Res.

[18] Kennedy RE, Williams CP, Sawyer P, Allman RM, Crowe M (2014). Comparison of in-person and telephone administration of the Mini-Mental State Examination in the University of Alabama at Birmingham Study of Aging. Journal of the American Geriatrics Society.

[19] McKhann GM (2011). The diagnosis of dementia due to Alzheimer’s disease: recommendations from the National Institute on Aging-Alzheimer’s Association workgroups on diagnostic guidelines for Alzheimer’s disease. Alzheimer’s & dementia.

[20] Albert MS (2011). The diagnosis of mild cognitive impairment due to Alzheimer’s disease: recommendations from the National Institute on Aging-Alzheimer’s Association workgroups on diagnostic guidelines for Alzheimer’s disease. Alzheimer’s & dementia: the journal of the Alzheimer’s Association.

[21] Molinuevo JL (2017). Implementation of subjective cognitive decline criteria in research studies. Alzheimers Dement.

[22] Jessen F (2014). A conceptual framework for research on subjective cognitive decline in preclinical Alzheimer’s disease. Alzheimers Dement.

[23] Dale AM, Fischl B, Sereno MI (1999). Cortical surface-based analysis. I. Segmentation and surface reconstruction. Neuroimage.

[24] Fischl B, Sereno MI, Dale AM (1999). Cortical surface-based analysis. II: Inflation, flattening, and a surface-based coordinate system. Neuroimage.

[25] Potvin O, Mouiha A, Dieumegarde L, Duchesne S (2016). Normative data for subcortical regional volumes over the lifetime of the adult human brain. Neuroimage.

[26] Potvin O, Dieumegarde L, Duchesne S (2017). Freesurfer cortical normative data for adults using Desikan-Killiany-Tourville and *ex vivo* protocols. Neuroimage.

[27] Potvin O, Dieumegarde L, Duchesne S (2017). Normative morphometric data for cerebral cortical areas over the lifetime of the adult human brain. Neuroimage.

[28] Coupe P (2008). An optimized blockwise nonlocal means denoising filter for 3-D magnetic resonance images. IEEE Trans Med Imaging.

[29] Sled JG, Zijdenbos AP, Evans AC (1998). A Nonparametric Method for Automatic Correction of Intensity Nonuniformity in MRI Data. IEEE Transactions on Medical Imaging.

[30] Collins DL, Neelin P, Peters TM, Evans AC (1994). Automatic 3D Intersubject Registration of MR Volumetric Data in Standardized Talairach Space. Journal of Computer Assisted Tomography.

[31] Fonov V (2011). Unbiased average age-appropriate atlases for pediatric studies. Neuroimage.

[32] Eskildsen SF (2012). BEaST: brain extraction based on nonlocal segmentation technique. Neuroimage.

[33] Pruessner JC (2002). Volumetry of temporopolar, perirhinal, entorhinal and parahippocampal cortex from high-resolution MR images: considering the variability of the collateral sulcus. Cereb Cortex.

[34] Pruessner JC (2000). Volumetry of hippocampus and amygdala with high-resolution MRI and three-dimensional analysis software: minimizing the discrepancies between laboratories. Cereb Cortex.

[35] Coupe P (2012). Simultaneous segmentation and grading of anatomical structures for patient’s classification: application to Alzheimer’s disease. Neuroimage.

[36] Nasreddine ZS (2005). The Montreal Cognitive Assessment, MoCA: a brief screening tool for mild cognitive impairment. J Am Geriatr Soc.

[37] Wechsler, D. *Wechsler memory scale - Fourth edition*. (The Psychological Corporation, 2009).

[38] Pachana NA (2007). Development and validation of the Geriatric Anxiety Inventory. Int Psychogeriatr.

[39] Yesavage JA (1988). Geriatric Depression Scale. Psychopharmacol Bull.

[40] Benjamini Y, Krieger AM, Yekutieli D (2006). Adaptive linear step-up procedures that control the false discovery rate. Biometrika.

[41] Jones, E., Oliphant, T. & Peterson, P. SciPy: Open Source Scientific Tools for Python. (2001).

[42] Seabold, S. & Perktold, J. Statsmodels: Econometric and statistical modeling with python. *Proceedings of the 9th Python in Science Conference* (2010).

[43] Iaccarino L (2018). Local and distant relationships between amyloid, tau and neurodegeneration in Alzheimer’s Disease. Neuroimage Clin.

[44] Bos I (2017). Cerebrovascular and amyloid pathology in predementia stages: the relationship with neurodegeneration and cognitive decline. Alzheimers Res Ther.

[45] Braak H, Braak E (1991). Neuropathological stageing of Alzheimer-related changes. Acta Neuropathol.

[46] Sun GZ (2017). Hippocampal synaptic and neural network deficits in young mice carrying the human APOE4 gene. CNS Neurosci Ther.

[47] Boyle PA (2017). Varied effects of age-related neuropathologies on the trajectory of late life cognitive decline. Brain.

[48] Adamowicz DH (2017). Hippocampal alpha-Synuclein in Dementia with Lewy Bodies Contributes to Memory Impairment and Is Consistent with Spread of Pathology. J Neurosci.

[49] Josephs KA (2017). Rates of hippocampal atrophy and presence of post-mortem TDP-43 in patients with Alzheimer’s disease: a longitudinal retrospective study. Lancet Neurol.

[50] Slavin MJ (2010). Prevalence and predictors of “subjective cognitive complaints” in the Sydney Memory and Ageing Study. Am J Geriatr Psychiatry.

[51] Stogmann E (2016). Activities of Daily Living and Depressive Symptoms in Patients with Subjective Cognitive Decline, Mild Cognitive Impairment, and Alzheimer’s Disease. J Alzheimers Dis.

[52] Jessen F (2014). AD dementia risk in late MCI, in early MCI, and in subjective memory impairment. Alzheimers Dement.

